# Effect of Vaginal Spheres and Pelvic Floor Exercises on Female Sexual Function in Women with Urinary Incontinence: A Randomized Controlled Trial (Secondary Analysis)

**DOI:** 10.1007/s00192-025-06404-7

**Published:** 2025-11-04

**Authors:** Laia Blanco-Ratto, Inés Ramirez-Garcia, Stephanie Kauffmann, Cristina Naranjo Ortiz, Montserrat Girabent Farrés

**Affiliations:** 1 RAPbarcelona Physiotherapy Clinical Center, Avinguda Diagonal 363, 3-2, Barcelona, 08037 Spain; 2 GHenderS Research Group, FCS Blanquerna- Ramon Llull University, Barcelona, Spain; 3 Blanquerna School of Health Science, Ramon Llull University, Barcelona, Spain; 4 Yale School of Medicine, Yale Cancer Center, Yale University, New Haven, CT USA; 5 Universitat de Vic - Universitat Central de Catalunya 22 (UVic-UCC), Campus Docent Sant Joan de Déu, Barcelona, Spain

**Keywords:** Physiotherapy, Pelvic floor exercises, FSFI, Female sexual dysfunction, Desire, Vaginal spheres, Pelvic floor muscle training

## Abstract

**Background:**

Female sexual dysfunction (FSD) is common in women with urinary incontinence (UI), and pelvic floor exercises (PFEs) are a well-established treatment. Vaginal spheres, designed to stimulate pelvic floor muscle engagement, may offer additional benefits in improving sexual function.

**Objective:**

To assess the efficacy of a regimen of PFEs combined with vaginal spheres with the same exercises performed without any device to improve sexual function.

**Methodology:**

This randomized, single-blind trial conducted at the RAPbarcelona Center enrolled 71 adult women with sexual dysfunction symptoms. Participants were assigned to either PFEs alone or combined with vaginal Enna Balls. The intervention lasted 4 months, with initial assessments at baseline and follow-ups at 8 and 16 weeks. The Female Sexual Function Index assessed sexual function. Adverse events and treatment adherence were monitored at weeks 4, 8, 12, and 16, and follow-ups.

**Results:**

The mean age of participants was 46.85±1.58. After treatment, no significant changes were observed in the overall Female Sexual Function Index score (≥0.05), except for an increase in desire, showing statistical differences between groups at the end of treatment (*p* = 0.041). Tolerance and safety did not significantly differ between groups.

**Conclusions:**

Adding vaginal spheres to PFEs may offer specific benefits in enhancing aspects of sexual function, particularly desire and lubrication, though overall outcomes were similar to PFEs alone. These findings support the potential role of vaginal spheres as an adjunctive therapy in personalized pelvic floor rehabilitation strategies for women with FSD.

## Introduction

Sexual health is a fundamental component of women’s quality of life, closely linked to psychological and social well-being [1]. Female sexual dysfunction (FSD) affects 19–50% of women and arises from physiological, psychological, and sociocultural factors, such as anatomical abnormalities, hormonal imbalances, pelvic floor disorders, and psychosocial stressors [1].

Pelvic floor muscle dysfunction is increasingly recognized as a major contributor to FSD. Research shows a strong correlation between FSD and pelvic floor muscle dysfunction (PFMD) [2]. These muscles support pelvic organs and influence arousal, orgasm, and vaginal tone. Their dysfunction can lead to pelvic organ prolapse, vaginal laxity, or impaired pelvic blood flow, all detrimental to sexual function [2].

Pelvic floor exercises (PFEs) are recommended to prevent and treat PFMD, enhancing arousal, satisfaction, and orgasm through improved muscle tone and blood flow [3]. PFEs also reduce symptoms like urinary incontinence and pelvic organ prolapse that interfere with sexual activity [1, 3].

Beji et al. showed PFEs benefit women with sexual dysfunction related to pelvic floor weakness or trauma [4]. More recent research has expanded on these findings, investigating the physiological and psychological mechanisms underlying the relationship between PFEs and sexual function. Jorge et al. [5] reported that women who regularly perform PEFs experience higher levels of sexual satisfaction, due to improved pelvic muscle strength and function, leading to increased vaginal tone and sensation during intercourse. This is particularly relevant for women with stress urinary incontinence (SUI), where PFEs both enhance sexual function and reduce leakage, a common source of sexual distress [3, 6].

PFEs can be used alone or with adjunct therapies such as electrical stimulation or biofeedback intravaginal medical devices like spheres or cones [7–9]. Despite the recognized benefits of PFEs, the role of adjunctive tools—such as vaginal spheres—remains underexplored [7, 10, 11]. Porta et al. (2010) previously investigated the use of vaginal spheres in pelvic floor rehabilitation [7, 12]; however, mid/long-term data on their efficacy and safety in the treatment of sexual dysfunction are lacking. Vaginal spheres, designed to facilitate muscle contraction during exercises, may improve training outcomes and sexual function [5, 7].

To address this gap in the literature, this secondary analysis compares PFEs alone vs. PFEs combined with vaginal spheres for FSD. We hypothesize that vaginal spheres will enhance outcomes in specific domains of the Female Sexual Function Index (FSFI) score and pelvic floor muscle strength.

## Materials and Methods

### Design

This single-blind, parallel randomized clinical study enrolled 71 women with sexual dysfunction recruited at RAPbarcelona Physiotherapy Clinical Center between October 2022 and May 2023 (ClinicalTrials.gov identifier NCT05732844). The study followed CONSORT guidelines [13].

### Participants

Participants were recruited consecutively at a specialized pelvic floor physiotherapy clinic. Before randomization, all participants received a comprehensive explanation of the study procedures, including potential risks and benefits, and provided written informed consent.

Eligible women were ≥18 years with stress or mixed urinary incontinence (SUI/MUI), per ICS criteria, assessed via the International Consultation on Incontinence Questionnaire Short Form Score (ICIQ-UI-SF) [14]. Sexual dysfunction was confirmed by self-reported symptoms during clinical interviews, focusing on desire, arousal, orgasm, satisfaction, and pain. A total score below 26.55 on the Female Sexual Function Index (FSFI) confirmed eligibility [15, 16].

Exclusion criteria included: (1) use of bladder-affecting medications (e.g., diuretics, anticholinergics); (2) stage III/IV prolapse; (3) BMI ≥30; (4) anal incontinence or complex UI; (5) pregnancy or <6 months postpartum; (6) chronic pelvic pain syndrome; and (7) prior PFE experience.

### Sample Size Calculation

Sample size calculation was based on Hirawaka et al., assuming a 4-point FSFI difference, SD 5, α = 0.05, F = 0.20 (80% power), and 10% attrition [17]. The final sample size was calculated accordingly.

### Allocation and Randomization

Participants were randomized 1:1 to either the intervention group (PFEs + vaginal spheres) or control group (PFEs only) using block randomization via Sealed Envelope Ltd. [18]. Allocation was concealed with sequential, opaque, stapled envelopes labeled with participant IDs. Envelopes were opened after baseline assessments to prevent allocation bias. The enrolling investigator was blinded to the sequence, and outcome assessors remained blinded to group allocation throughout the study to minimize assessment bias.

### Ethical considerations

The study adhered to the Declaration of Helsinki and was approved by Vall d’Hebron Hospital Ethics Committee (PR(RAP)402/2019). Written informed consent was obtained from all participants.

### Intervention

This study utilized vaginal spheres (Enna Balls, ECARE YOU, SL. Barcelona, Spain), each weighing 0.13 grams with a height of 0.103 m and an amplitude of 0.073 m. The intervention spanned 16 weeks and was conducted at home according to the following regimen:

- Weeks 1–2: 15 min/day
- Weeks 2–4: 30 min/day
- Weeks 4–8: 1 h/day
- Weeks 8–12: 2 h/day
- Weeks 12–16: 3 h/day

Participants received instruction on hygiene and insertion (e.g., identifying the vaginal canal, full insertion for comfort). Lubricants were limited to water-based options.

### Pelvic Floor Exercise Regimen

Participants in the intervention group also performed 48 daily pelvic floor exercises (PFEs), consisting of:

- slow contractions (4–10 s, progressively increasing over 16 weeks).
- 8 fast contractions (1–2 s).
- A 2-minute rest between sets.

Each set of 16 contractions was repeated three times daily, totaling 48 contractions per day, 7 days a week, for 4 months. Exercises were performed in different positions throughout the day:

- Morning: Lying down
- Midday: Semi-recumbent
- Afternoon: Standing

This regimen followed the protocol described by Dayican et al. [19].

### Control Group

The control group followed the same PFE protocol without the use of vaginal spheres.

### Supervision and Assessment

All participants, including those with an Oxford score of 0, received individualized training by pelvic floor physiotherapists. Assessments were performed at baseline and weeks 4, 8, 12, and 16, including digital palpation and Oxford Scale grading. Physiotherapists received standardized training to ensure protocol consistency.

### Outcome Measures

Female sexual function and urinary incontinence were assessed using validated Spanish versions of the FSFI and the ICIQ-UI-SF questionnaire, respectively.

The FSFI is a widely used, self-reported questionnaire designed to evaluate female sexual function across multiple domains, including desire, arousal, lubrication, orgasm, satisfaction, and pain. Developed by Rosen et al. [15], it has demonstrated robust psychometric properties and is frequently utilized in both clinical and research settings for assessing sexual dysfunction. The FSFI has been validated across diverse populations, including women with conditions such as diabetes, cancer, and pelvic organ prolapse. While it has been extensively used in Spain, culturally adapted validations specific to the Spanish population have only recently been published [20].

The FSFI consists of 19 items, each scored on a Likert scale from 0 to 5, with higher scores indicating better sexual function. Domain scores are summed and adjusted using specific multipliers to ensure a balanced presentation. The total FSFI score ranges from 2 to 36, with a commonly accepted clinical threshold for sexual dysfunction being a total score of <26.55 [15, 16].

Assessments were conducted at baseline and two follow-up points (weeks 8 and 16 post-treatment initiation).

The ICIQ-UI-SF is a validated instrument designed to assess UI severity and its impact on quality of life. This Spanish-validated questionnaire [21] has four items evaluating incontinence frequency, severity, and associated life impact. It is widely used in clinical and research settings and is recommended by international guidelines, including the ICS [14].

The ICIQ-UI-SF is scored on a numerical scale, with higher scores indicating greater incontinence severity. The final score (range 0–21) categorizes UI severity [14, 21].

- **Mild:** 1–5 (infrequent leakage with minimal impact on daily life).
- **Moderate:** 6–12 (more frequent leakage with moderate daily life impact).
- **Severe:** 13–21 (frequent or large-volume leakage with significant quality-of-life impairment).

Assessments were conducted at baseline and at the end of the treatment period.

Pelvic floor muscle strength was evaluated using the Oxford Grading Scale, a widely used and validated tool for assessing muscle contraction strength through digital palpation. The scale categorizes muscle contraction as follows:

- **Grade 0:** No contraction.
- **Grade 1:** Flicker contraction.
- **Grade 2:** Weak contraction.
- **Grade 3:** Moderate contraction.
- **Grade 4:** Strong contraction.
- **Grade 5:** Full contraction (sustained and controlled).

A trained examiner assessed pelvic floor muscle strength using digital palpation at baseline and follow-up visits, following standardized methodologies [22].

Adverse events were systematically documented after each treatment session. Treatment adherence was assessed using an adapted version of the Morisky–Green test, in which “taking medication” was replaced with “performing pelvic floor exercises,” as previously validated by Porta et al. [7, 12].

### Data Analysis

Data normality was assessed with the Kolmogorov–Smirnov test. As normality was not confirmed, between-group comparisons at baseline, week 8, and week 16 were conducted with the Mann–Whitney U test. Results are reported as means (SD) and medians (IQR). Between-group differences were estimated with the Hodges–Lehmann median difference and 95% CI, and effect sizes were quantified with Cliff’s delta (δ), interpreted as negligible (∣δ∣<0.11|δ|<0.11∣δ∣<0.11), small (0.11–0.28), medium (0.28–0.43), or large (∣δ∣≥0.43|δ|≥0.43∣δ∣≥0.43).

Analyses followed the per-protocol approach; an intention-to-treat (ITT) analysis with multiple imputation for missing data was also performed. Significance was set at *p* < 0.05. Analyses were performed using SPSS v29.0 (IBM Corp., Chicago, IL).

Data utilized in this study are publicly available at Harvard Dataverse (10.7910/DVN/DVOHPZ).

## Results

### Study Enrollment and Participant Flow

**Fig. 1:**
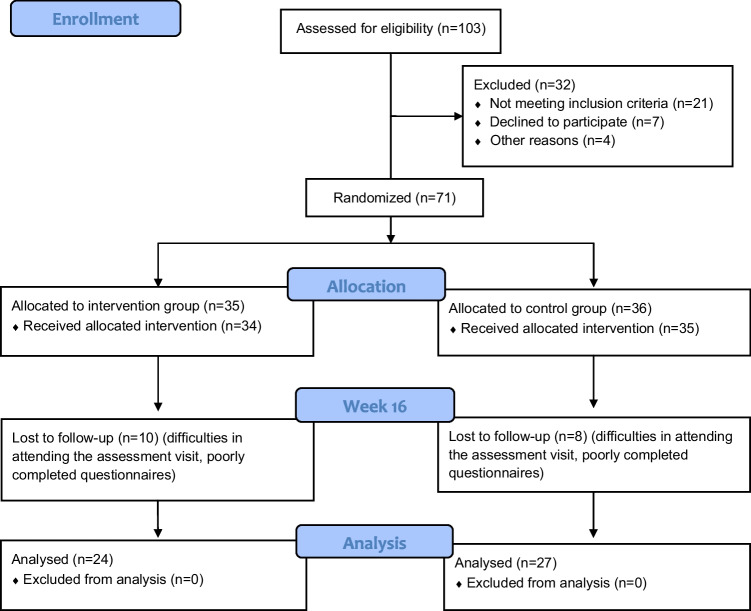
CONSORT participant flow diagram

Of the 103 eligible participants, 71 met the inclusion criteria. The remaining 32 were excluded due to medical conditions (*n* = 21), refusal to participate (*n* = 7), or psychological concerns such as fear of using vaginal spheres or concerns about infection risk (*n* = 4). A detailed flowchart is presented in Fig. 1.

Of the 71 included participants, 35 were randomized to the PFEs with vaginal spheres group and 36 to the PFEs alone group. By week 8, data from 69 patients (34 in the intervention group and 35 in the control group) were available for analysis. Only two participants (one from each group) dropped out after the initial consultation.

By week 16, complete data were available for 51 participants. Eleven participants in the intervention group and nine in the control group were lost to follow-up due to difficulty attending visits or incomplete questionnaire responses.

A descriptive analysis was performed to examine the reasons for dropout, and baseline characteristics were compared between those who completed the study and those who discontinued, demonstrating no significant differences between groups.

### Adherence to the Trial Protocol

To promote adherence, participants received regular reminders via phone or email 24 h before scheduled sessions. Missed sessions were promptly rescheduled to minimize disruption. Follow-up visits provided additional guidance and progress assessments.

Adherence was assessed using self-reported data (adapted Morisky–Green test) and objective assessments by a physiotherapist during follow-up visits. Participants who failed to meet the predefined adherence thresholds, such as consistent vaginal sphere use and completion of at least 70% of prescribed exercises (equivalent to a minimum of 15 sessions per week) were excluded from the final analysis.

All prespecified outcome measures are reported in this manuscript.

### Baseline Characteristics

Baseline demographic and clinical characteristics were compared using the Mann-Whitney U-test for continuous variables and the chi-square or Fisher’s exact test for categorical variables. As shown in Table 1, no significant differences were observed between groups.

**Table 1:** Baseline clinical characteristics of control and intervention groups

	Group (*n* = 69)				*p*-value^a^	CI 95%	
	Control group (*n* = 35)		Intervention (*n* = 34)				
	Md (IQR)	Mean (SD)	Md (IQR)	Mean (SD)		Lower	Upper
Age	40.00 (34.00–57.00)	45.63 (13.23)	50.00 (35.00–59.00)	48.12 (13.07)	0.364	−8.00	2.00
UI symptoms duration	27.00 (8.00–84.00)	63.88 (108.00)	24.00 (10.00–72.00)	76.79 (122.44)	0.930	−18.00	15.00
Full-term pregnancies	1.00 (1.00–2.00)	1.37 (0.81)	2.00 (1.00–2.00)	1.59 (0.89)	0.303	−1.00	0.00
Menopause age	50.00 (42.00–56.00)	49.67 (3.96)	51.00 (43.00–55.00)	49.89 (3.68)	0.426	−6.00	1.00
ICIQ-UI-SF	8.00 (6.00–12.00)	9.00 (4.44)	8.00 (6.00–13.00)	9.03 (4.34)	0.895	−2.00	2.00
Desire	2.40 (1.80–3.60)	2.57 (1.06)	3.00 (2.40–3.60)	2.88 (1.28)	0.382	−1.20	0.00
Arousal	3.60 (2.40–4.80)	3.34 (1.84)	3.90 (2.10–4.80)	3.40 (1.99)	0.796	−0.90	0.60
Lubrication	3.90 (2.70–5.40)	3.71 (1.94)	3.90 (2.40–4.80)	3.25 (1.84)	0.160	−0.30	1.20
Orgasm	4.00 (2.40–5.20)	3.65 (2.08)	4.40 (2.80–5.60)	3.86 (1.99)	0.646	−1.20	0.80
Satisfaction	4.40 (2.40–5.20)	3.85 (1.69)	4.00 (2.40–5.20)	3.69 (1.72)	0.717	−0.40	0.80
Pain	4.40 (1.60–5.60)	3.58 (2.28)	4.20 (2.00–5.60)	3.62 (2.26)	0.947	−0.80	0.80
FSFI score	19.40 (15.90–22.60)	18.06 (7.00)	20.40 (14.70–25.80)	19.08 (7.86)	0.401	−4.40	1.80
Oxford power	2.00 (1.00–2.00)	1.77 (0.91)	2.00 (1.00–2.00)	1.82 (0.83)	0.975	0.00	0.00

*n* number of patients, *Md* Median, *IQR* Interquartile range, *SD* Standard deviation, *UI* Urinary incontinence, *ICIQ-UI-SF* International Consultation on Incontinence Questionnaire Short Form, *FSFI* Female Sexual Function Index. Mann–Whitney U test, *CI* Confidence interval

None of the control group participants received hormone replacement therapy, while one in the intervention group did. Over 80% of participants in both groups were not taking any medication; seven participants (three control, four intervention) received topical hormone treatment.

In terms of urinary incontinence (UI) severity, 68.6% of the control group and 55.9% of the intervention group had mild incontinence; 25.7% and 38.2% reported moderate incontinence, respectively. Severe UI was seen in 5.7% (control) and 5.9% (intervention). These differences were not statistically significant (*p* =.523).

Similarly, no significant difference was observed in the type of incontinence (*p* =.103): 77.1% of the control group participants and 58.8% of the intervention group had stress urinary incontinence (SUI).

Baseline FSFI, ICIQ-UI-SF, and Oxford Scale scores were also comparable (Table 1).

### Intervention Outcomes

At week 8, 69 participants who completed the FSFI, ICIQ-UI-SF, and Oxford Scale assessments were included in the per-protocol (PP) analysis. By week 16, 51 participants (24 in the intervention group and 27 in the control group) completed all assessments.

- Female Sexual Function: After 16 weeks of PFEs (with or without vaginal spheres), significant improvements were observed in multiple FSFI domains. The intervention group showed statistically significant mean increases in desire (+0.65, *p* =.047), arousal (+0.84, *p* =.014), and lubrication (+0.82, *p* =.026) (Tables 2 and 3 present the changes in FSFI questionnaire scores over time). The control group exhibited significant improvements in arousal at week 8 (+0.30, *p* <.000) and pain reduction at week 16 (−0.57, *p* =.045).

**Table 2:** Values of ICIQ-UI-SF, FSFI, and Oxford power at week 8

	Group (*n* = 69)				*p*-value^a^	CI 95%		Effect size	
	Control group (*n* = 35)		Intervention (*n* = 34)						
	Md (IQR)	Mean (SD)	Md (IQR)	Mean (SD)		Lower	Upper	Cliff’s delta	Interpretation
ICIQ-UI-SF	5.00 (4.00–9.00)	6.49 (4.09)	5.00 (3.00–6.00)	5.56 (4.18)	0.355	−1.00	3.00	−0.129	Small
Desire	2.40 (2.40–3.60)	2.73 (1.04)	3.60 (2.40–4.20)	3.18 (1.31)	0.076	−1.20	0.00	0.243	Small
Arousal	3.90 (2.70–5.10)	3.64 (1.81)	4.35 (2.40–5.10)	3.54 (2.14)	0.875	−0.90	0.90	0.022	Trivial
Lubrication	4.50 (3.00–5.10)	3.75 (1.90)	4.50 (1.20–5.10)	3.44 (2.13)	0.708	−0.60	0.90	−0.052	Trivial
Orgasm	4.40 (2.40–5.60)	3.77 (2.10)	4.80 (2.80–5.60)	3.84 (2.13)	0.809	−0.80	0.80	0.034	Trivial
Satisfaction	4.80 (2.40–5.20)	3.82 (1.89)	4.40 (1.60–5.20)	3.56 (2.12)	0.707	−0.40	0.80	−0.052	Trivial
Pain	5.20 (1.60–6.00)	3.86 (2.42)	4.40 (0.00–6.00)	3.46 (2.57)	0.486	0.00	1.20	−0.095	Trivial
FSFI Score	20.10 (16.40–22.70)	18.75 (6.89)	19.35 (14.30–24.00)	18.43 (8.59)	0.792	−3.10	3.40	−0.037	Trivial
Oxford power	2.00(2.00–3.00)	2.31 (0.83)	2.50 (2.00–3.00)	2.44 (0.61)	0.349	−1.00	0.00	0.120	Small

*n* Number of patients; *Md* Median; *IQR* Interquartile range; *SD* Standard deviation; *ICIQ-UI-SF* International Consultation on Incontinence Questionnaire Short Form; *FSFI:* Female Sexual Function Index; Mann–Whitney U test; *CI* Confidence interval

**Table 3:** Values of ICIQ-UI-SF, FSFI, and Oxford power at the end of treatment

	Group (*n* = 51)				*p*-value^a^	CI 95%		Effect size	
	Control group (*n* = 27)		Intervention (*n* = 24)						
	Md (IQR)	Mean (SD)	Md (IQR)	Mean (SD)		Lower	Upper	Cliff’s delta	Interpretation
ICIQ-UI-SF	4.00 (0.00–8.00)	4.52 (4.85)	3.50 (0.00–5.00)	3.31 (3.69)	0.442	0.00	1.36	−0.045	Trivial
Desire	3.00 (2.40–3.60)	2.89 (1.15)	3.60 (2.40–4.20)	3.53 (1.25)	0.041*	**−0.99**	**−0.60**	**0.483**	**Large**
Arousal	4.50 (3.00–5.40)	3.81 (2.02)	5.10 (3.30–5.40)	4.24 (1.74)	0.361	−0.60	0.00	0.287	**Medium**
Lubrication	4.8 (2.40–5.10)	3.69 (2.10)	4.50 (3.60–5.40)	4.07 (1.81)	0.596	−0.60	0.00	0.219	Small
Orgasm	4.80 (3.20–5.20)	3.91 (2.12)	5.20 (3.60–5.60)	4.33 (1.81)	0.474	−0.40	0.00	0.252	Small
Satisfaction	4.8 (3.60–5.20)	4.27 (1.68)	4.80 (3.20–5.20)	4.15 (1.60)	0.681	0.00	0.40	0.052	Trivial
Pain	5.60 (0.00–6.00)	4.15 (2.54)	5.20 (0.00–6.00)	4.00 (2.49)	0.665	0.00	0.25	0.051	Trivial
FSFI Score	19.40 (16.90–21.70)	18.35 (5.47)	18.20 (15.20–23.30)	19.00 (7.10)	0.869	–1.50	0.90	0.014	Trivial
Oxford power	3.00 (3.00–3.00)	2.93 (0.68)	3.00 (3.00–3.00)	2.96 (0.6)	0.814	0.00	0.00	0.116	Small

*n* Number of patients, *Md* Median, *IQR* Interquartile range, *SD* Standard deviation, *ICIQ-UI-SF* International Consultation on Incontinence Questionnaire Short Form, *FSFI* Female Sexual Function Index, Mann–Whitney U test, *p value < 0.05; *CI* Confidence interval

**Fig. 2:**
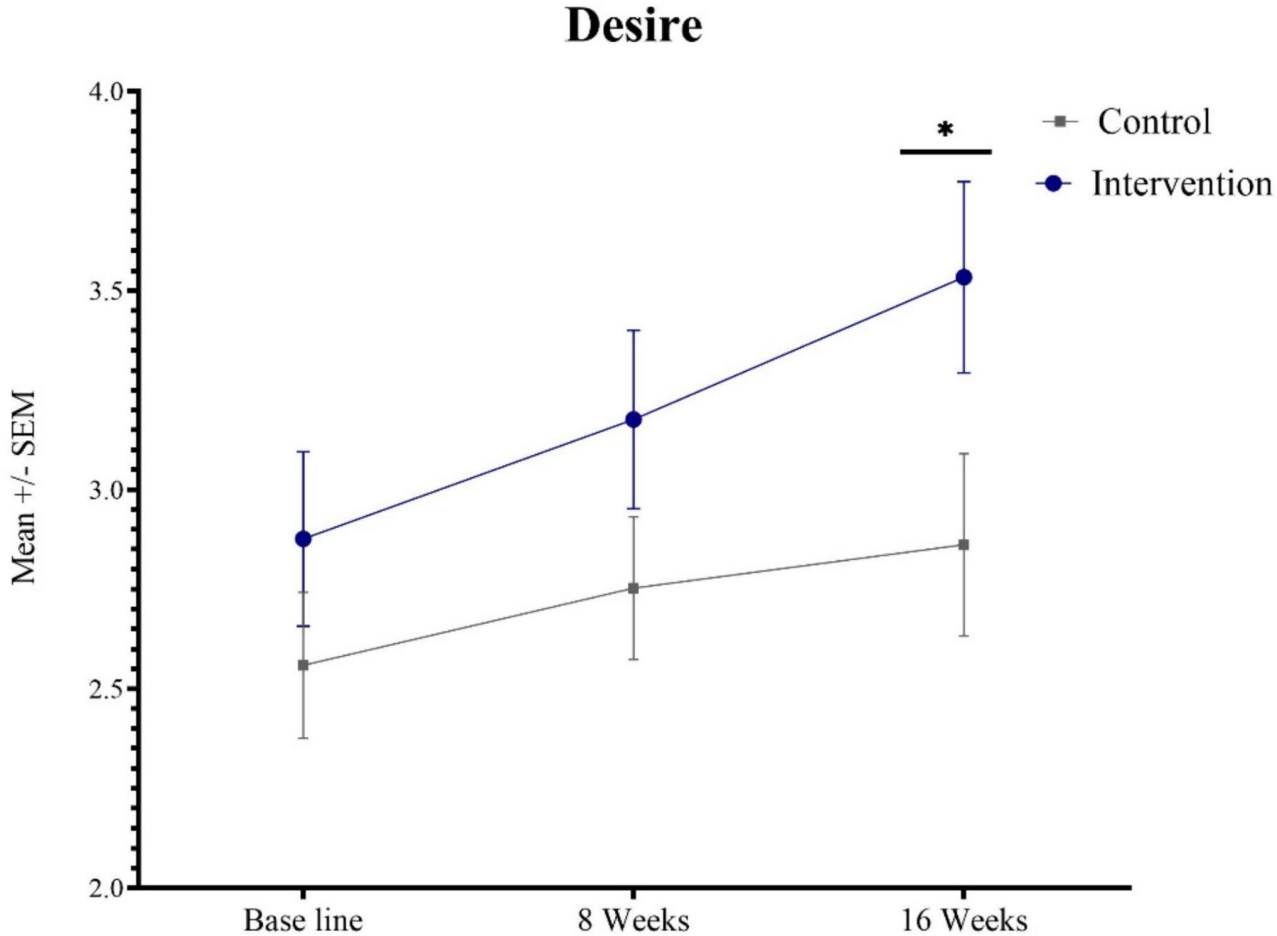
Changes in desire scores over time

Despite these within-group improvements, no significant differences were observed between groups in total FSFI scores at the end of treatment (*p* =.869). Similarly, no significant between-group differences were found for arousal (*p* =.361), lubrication (*p* =.596), orgasm (*p* =.474), satisfaction (*p* =.681), or pain (*p* =.665). However, a statistically significant between-group difference in sexual desire emerged at week 16 (c.041) (Fig. 2).

- Urinary Incontinence: Both groups experienced significant improvements in ICIQ-UI-SF scores over time, with reductions of 4.48 points in the control group (*p* <.000) and 5.72 points in the spheres group (*p* <.000). However, no statistically significant differences were observed between groups at either week 8 (*p* =.355) or week 16 (*p* =.442) (Tables 2 and 3).
- Pelvic floor strength: Per-protocol analysis demonstrated significant within-group improvements in pelvic floor muscle strength, as measured by the Oxford Scale. The control group improved by 1.16 points (*p* <.000), while the intervention group improved by 1.14 points (*p* <.000). No significant between-group differences were observed (*p* =.814) (Tables 2 and 3).

To evaluate the robustness of the results, an intention-to-treat (ITT) analysis was conducted using multiple imputation to address missing data, generating five imputed datasets. Across all imputations, the variable “desire” consistently showed a statistically significant difference between the intervention and control groups, with *p* values of 0.010, 0.015, 0.020, 0.049, and 0.030, respectively. For all other outcome variables, no significant differences were observed across the imputations (all *p* values > 0.05), supporting the consistency and robustness of the findings.

### Patient Tolerance Assessment

Subjective tolerance was assessed throughout the study. By week 4, a statistically significant reduction in discomfort was observed (*p* =.005). No participants in the control group reported discomfort at this time, whereas 20.6% of the intervention group experienced some discomfort due to the vaginal spheres. By week 8, discomfort was reported by 3% of the control group and 11% of the intervention group (*p* =.174). By week 12, discomfort persisted in 2.9% of the control group and 15.2% of the intervention group (*p* =.074). At week 16, no participants in the control group reported discomfort, while 11.5% of the intervention group still experienced minor discomfort. No study withdrawals were attributed to adverse effects.

#### Adherence Assessment

Adherence was assessed using the adapted Morisky–Green test. At week 4, 80% of participants in the control group reported missing exercise sessions (at least three exercise sessions per week), compared with 50% in the intervention group. A similar trend persisted at week 8 (71.4% vs. 52.9%) and week 12 (74.3% vs. 54.5%), and by week 16 the proportion remained higher in the control group (81.5%) than in the vaginal spheres group (50%). All participants included in the per-protocol analysis used the vaginal spheres for the prescribed duration; the main adherence difficulty related to completing all daily exercise sets rather than device use. Although statistical significance was not achieved at all time points, the consistent pattern suggests that vaginal spheres may support better adherence to pelvic floor training. This adherence difference was statistically significant (*p* <.05 during all weeks.

In summary, both PFEs alone and PFEs with vaginal spheres significantly improved sexual function, urinary incontinence, and pelvic floor muscle strength over 16 weeks. The intervention group showed more significant improvements in sexual desire and adherence, although no significant differences in overall FSFI, ICIQ-UI-SF, or Oxford Scale scores were observed between groups. The intervention was well-tolerated, with minor discomfort reported by a small percentage of participants. Adherence was significantly higher in the intervention group, suggesting that the addition of vaginal spheres may enhance compliance with pelvic floor exercise regimens.

## Discussion

This study evaluated whether adding vaginal spheres to PFEs enhances sexual function in women with urinary incontinence. We hypothesized that adding vaginal spheres would lead to greater improvements in sexual function compared to PFEs alone.

Our findings partially support this hypothesis, demonstrating improvements in sexual desire, arousal, and lubrication in the intervention group. Notably, sexual desire showed the most pronounced enhancement at week 16 in the vaginal spheres group compared to the control. These results suggest that vaginal spheres may provide additional benefits beyond PFEs alone, aligning with previous research on the role of pelvic floor rehabilitation in sexual function improvement [2–4, 6].

### Mechanisms Underlying the Improvement in Sexual Function

The observed improvement in sexual desire in the vaginal spheres group may be attributed to several physiological and psychological mechanisms. Vaginal spheres are designed to promote pelvic floor muscle engagement, enhancing muscle tone, vaginal tightness, and sensory perception, which are crucial for sexual arousal and pleasure [7, 12]. Additionally, increased blood flow to the pelvic region may facilitate vaginal lubrication, contributing to overall sexual function enhancement [23]. Psychological factors may also play a role; the use of vaginal spheres may boost self-confidence and body image, both of which are closely linked to sexual desire and satisfaction [24]. Given the multidimensional nature of sexual desire, these combined effects likely contributed to the improvements observed in the intervention group.

An additional factor to consider is the hormonal profile of our participants. Most women in the study were peri-menopausal, and only a minority were receiving local estrogen or hormone replacement therapy. This may have influenced both responsiveness to pelvic floor muscle training and tolerance of vaginal spheres. The presence of genitourinary syndrome of menopause, with associated vaginal dryness and atrophy, could explain the transient discomfort reported by some participants, while hormonal changes may also affect muscle contractility and rehabilitation outcomes. Future studies should stratify participants by hormonal status and evaluate whether the adjunctive use of local estrogen or vaginal moisturizers improves both treatment tolerability and efficacy.

### FSFI Outcomes and Clinical Implications

Despite significant improvements in specific domains of sexual function, no significant difference was observed in overall FSFI scores between groups. This suggests that while vaginal spheres may enhance select aspects of sexual function, their overall impact may not be substantial enough to surpass the therapeutic effects of PFEs alone. PFEs are well-documented as effective interventions for urinary incontinence and sexual dysfunction [2, 3], and the lack of a spheres-only control group in our study may have limited the ability to detect significant between-group differences. Future studies excluding PFEs may help clarify the independent contribution of vaginal spheres to sexual function improvement.

The FSFI desire domain serves as an independent measure of sexual function, with a score threshold of 5 used for diagnosing Hypoactive Sexual Desire Disorder (HSDD) [25]. Although the mean FSFI desire score in the intervention group remained below this diagnostic threshold, the consistent and statistically significant improvement observed across both per-protocol and intention-to-treat analyses may still represent a clinically meaningful change. Incremental gains in sexual desire have been linked to improved sexual well-being, greater motivation to engage in sexual activity, and reduced sexual distress, particularly in perimenopausal women.

Furthermore, these improvements should be interpreted in the context of multi-domain changes. The simultaneous gains in desire, arousal, and lubrication suggest that vaginal spheres may enhance multiple aspects of sexual response. Taken together, these effects may contribute to greater patient satisfaction, adherence to therapy, and overall quality of life, even if diagnostic thresholds for HSDD are not fully surpassed.

### Early Changes in Sexual Arousal and Pain Reduction

Interestingly, an improvement in sexual arousal was observed in the control group as early as week 8. This early response may be attributed to the well-established benefits of PFEs, including enhanced blood flow, muscle tone, and nerve sensitivity in the pelvic region. Additionally, a significant pain reduction was observed in the control group by the end of treatment. The interplay between arousal and pain is well documented, as reduced discomfort during intercourse often enhances sexual satisfaction and arousal [26]. PFEs alleviate dyspareunia by strengthening pelvic muscles, reducing tension, and increasing lubrication. Thus, the observed pain reduction over time likely contributed to the improvement in sexual arousal, underscoring the importance of pelvic rehabilitation in managing sexual dysfunction [2, 4–6, 27, 28].

Overall, our results indicate that both pelvic floor exercises with vaginal spheres and PFEs alone can positively influence female sexual function. However, the specific benefits of vaginal spheres may be more evident in certain domains, such as sexual desire and lubrication. Future research with larger sample sizes and more extended follow-up periods is needed to better understand the long-term effects of vaginal spheres in combination with PFEs and to further explore the mechanisms underlying the observed improvements in sexual function. Furthermore, understanding the interplay between physical and psychological factors in the context of pelvic floor rehabilitation will be essential for optimizing treatment strategies for female sexual dysfunction.

### Pelvic Floor Strength and Changes in Incontinence

Our results indicate that both intervention and control groups experienced significant improvements in UI, as reflected by reductions in ICIQ-UI-SF scores. However, no significant between-group differences were observed, suggesting that vaginal spheres did not prove additional benefits over PFEs alone in addressing urine leakage. These findings align with prior studies demonstrating that PFEs are highly effective for incontinence management, while the addition of vaginal devices does not necessarily enhance these outcomes [7, 29].

Both groups exhibited statistically significant improvements over the study period with similar increases on the Oxford Scale. The vaginal spheres group showed a 1.14-point improvement on the Oxford Scale, while the control group demonstrated an increase of 1.16 points. Interestingly, while the strength improvements were similar between both groups, the potential for vaginal spheres to enhance PFMT’s effectiveness on sexual function, specifically desire, may offer a distinct advantage.

### Safety and Tolerance of Vaginal Spheres

Our study confirms that the combination of PFEs and vaginal spheres is safe and well-tolerated, with tolerance improving throughout the study in both groups. Seven participants reported mild adverse effects at week 4 (visit 2). Specifically, one patient in the control group experienced low tolerance to physical therapy, reporting pelvic floor pain. In contrast, four participants in the vaginal spheres group reported hypersensitivity, irritation, itching, and local discomfort. However, these adverse effects had subsided or diminished in all patients by the next follow-up visit, indicating a proper adaptation to the physical therapy methods. The control group also reported one case of low tolerance to PFEs, reinforcing the need for individualized patient guidance in pelvic floor rehabilitation programs.

Interestingly, adherence was significantly higher in the vaginal spheres group. Although no formal qualitative data were collected, informal feedback during follow-up visits suggested that women perceived the spheres as a tangible aid that provided sensory cues and enhanced motivation, potentially reducing the monotony of repeated pelvic floor exercises. The novelty and perceived benefit of the device may therefore have contributed to better adherence, aligning with previous reports that adjunctive intravaginal devices can improve compliance by increasing engagement and perceived therapeutic value [3, 10, 12].

It is worth noting that these mild discomforts may have been less pronounced in a non-perimenopausal population, as observed in previous studies such as the one by Porta et al. (2015) [7]. The participants in our study were peri-menopausal, with an average age of 46.85±1.58 years, and more likely to experience vaginal atrophy associated with the genitourinary syndrome of menopause. Additionally, only one participant in the study was undergoing hormone replacement therapy, and only seven women (three in the control group and four in the intervention group) were using topical hormone treatments. From a clinical standpoint, it would be beneficial to consider including topical hormone treatments or the combined use of vaginal moisturizers in future studies to prevent irritation and discomfort due to contact with atrophic mucosa.

## Limitations and Future Directions

Several limitations should be acknowledged. One key limitation is that PFEs were included in both groups, which prevents us from isolating the independent effects of vaginal spheres to sexual function outcomes. This design choice was guided by ethical considerations, as PFEs are first-line, evidence-based treatment for UI and SD and could not reasonably be withheld from participants. However, this limits the ability to determine whether the observed improvements in desire, arousal and lubrication were due to vaginal spheres alone or the synergistic effect of combining them with PFEs. Future research should address this by including a vagina-spheres only group or adopting a three-arm design (PFEs alone vs. vaginal spheres alone vs. PFEs + vaginal spheres), allowing for a clearer interpretation of both independent and combined effects.

Another limitation is the relatively short follow-up period of 16 weeks, which does not allow determination of the long-term sustainability of the observed improvements in sexual function. Future studies with longer follow-up durations are needed to evaluate the durability and clinical relevance of these effects.

A higher-than-expected loss to follow-up (28%) represents an additional limitation, as it may have reduced statistical power and introduced attrition bias. Although baseline characteristics were comparable between completers and non-completers, differential dropout cannot be ruled out, and results should therefore be interpreted with caution.

Additional limitations include the absence of an inactive control group, which further limits the ability to isolate the effects of vaginal spheres. Further research should explore the interplay between physiological and psychological factors in sexual function rehabilitation, as addressing both domains may optimize treatment outcomes.

## Conclusion

In conclusion, both PFEs alone and in combination with vaginal spheres can improve sexual function and urinary incontinence in peri-menopausal women; the addition of vaginal spheres may offer targeted benefits, particularly in enhancing sexual desire, arousal, and lubrication. Despite the lack of significant between-group differences in overall FSFI scores and incontinence outcomes, the domain-specific improvements observed in the intervention group suggest a potential role for vaginal spheres as a complementary tool in pelvic floor rehabilitation. These findings underscore the importance of individualized approaches to female sexual dysfunction, particularly in peri-menopausal populations. Future research should investigate the long-term effects of combined therapies, optimize treatment protocols, and evaluate the role of adjunctive treatments such as topical estrogen in addressing genitourinary atrophy and improving treatment tolerability.

## Data Availability

Data utilized in this study are publicly available at Harvard Dataverse (10.7910/DVN/DVOHPZ).
